# Migraine and Current Pharmacologic Management

**DOI:** 10.7759/cureus.29833

**Published:** 2022-10-02

**Authors:** Okelue E Okobi, Maureen G Boms, Joseph C Ijeh, Stephen E Eboigbe, Kesena B Alex, Adebisi A Adejola, Uduak A Udo, Donnee Athem, David Oboh, Eniola Olamilehin, Oyintoun-emi Ozobokeme, Adeoluwa Adegbosin, Lucy Nwaeke, Endurance O Evbayekha

**Affiliations:** 1 Family Medicine, Lakeside Medical Center, Belle Glade, USA; 2 Clinical Research, The University of Alabama at Birmingham, Birmingham, USA; 3 Family Medicine, Gennesaret Medical Center, Laurel, USA; 4 General Practice, Lagos University Teaching Hospital, Lagos, NGA; 5 Medicine, University of London, London, GBR; 6 General Internal Medicine, The Meriden Hospital, Coventry, GBR; 7 Radiology, Imaging for Women, Kansas City, USA; 8 Internal Medicine, Igbinedion University Okada, Okada, NGA; 9 Radiology, University College Hospital, Ibadan, NGA; 10 Internal Medicine, St. George's University School of Medicine, St. George's, GRD; 11 Medicine, Central Michigan University College of Medicine, Mount Pleasant, USA; 12 Psychiatry and Behavioral Sciences, Kharkiv National University, Kharkiv, UKR; 13 Internal Medicine, Washington University of Health and Science, San Pedro, BLZ; 14 Internal Medicine, St. Luke's Hospital, St. Louis, USA

**Keywords:** headache disorders, chronic migraine (cm), resistant migraine, migraine disorder, migraine

## Abstract

In light of the multiple pharmacologic alternatives for migraine management, the adverse effects associated with different treatment regimens, and their varying efficacy, it is vital to undertake ongoing evaluations and seek a tailored therapeutic strategy. This case report describes two patients who presented with classic migraine headaches, with the first responding to both abortive and prophylactic migraine medications and the second, with status migrainosus-like presentation, responding to the newer antimigraine agents. We also examined current pharmacological therapeutic options and existing recommendations, analyzing their strengths and weaknesses. This case review exemplifies how treatment in the last 10 years and success in medications used for abortive and prophylactic therapy are evolving from the older agents to newer agents like the recently approved calcitonin gene receptor protein monoclonal antibody inhibitors.

## Introduction

Many people often term recurrent occurrences of headaches to be migraine. Migraine is a primary headache disorder, often manifesting as an acute or chronic neurological condition [1]. Several schools of thought have classified this headache as pulsating, throbbing pain with varied efforts to construct consistently accepted diagnostic criteria. The International Society of Migraines described this headache as recurring with at least five episodes that persist for at least four to 72 hours when untreated or undertreated [1-4]. The American Headache Society is also in synchrony with the definition of this type of headache to be more than five attacks to include characteristics like unilaterality of pain, pulsation, aggravating factors like walking, as well as photophobia, phonophobia, nausea, and/or vomiting that must be present during the episodes of attack in the absence of any other etiological explanation [5,6]. In addition, in one of their latest update in 2020, the European Headache Federation classified it as refractory or resistant migraine [1-7]. The International Classification of Diseases, Tenth Revision (ICD-10) classification of this headache includes migraine with aura, migraine without aura, status migrainosus, complicated migraine, and other types of migraine [1-4,8]. Aura is a complex neurological motor, sensory, or visual manifestation that appears a few minutes before or concurrently with the headache and lasts up to an hour with or after the headache [8-15]. Some migraine has also been associated with prodromal phases, pre, with, or post-head, with common prodromal symptoms characterized by neck stiffness, neck pain, food craving, hypo or hyperactivity, depression, and repetitive yawning [1-6,11-15]. Epidemiologically, migraine has been described as amongst the world's third most common illnesses and the leading cause of disability in people aged 50 years or younger [5-10]. With an estimated lifetime prevalence of 16% in the general population [9-11], migraine is the most common class of headaches encountered in persons seeking care for a headache, with a peak incidence between the 20s and mid-50s in age; females are twice as likely as males to be afflicted, while boys in the pre-pubertal period are more likely to be affected [4-11]. The role of genetics has been reported, with a significant percentage of migraine sufferers having a first-degree relative with migraine [2,4-15].

The exact cause of migraine is unknown [1,2,4-12], but it is thought to be caused by a complex cascade of neurovascular disorders in the cranial nerve pain pathway, particularly the involvement of trigeminovascular neurons, issues with hypersensitivity of gated channels, and abnormal processing of signal transmission from perivascular neurons to the meninges or overexcitation of the brain's stem pain centers involving endogenous neurotransmitters such as calcitonin gene-related peptide (CGRP), substance P, pituitary adenylate cyclase-activating peptide, neurokinin A, and other vasoactive peptides [1,2,4-6,9-15]. These next-generation sequencing-based studies have accelerated the discovery of single locus mutations and mendelian inheritance patterns in the genes of people with some rare forms of inherited migraine variants such as familial migraine and hemiplegic migraine, particularly genes like SCN1A, CACNA1A, and ATP1A2 [15-17], Furthermore, the diagnosis of migraine is made after excluding other causes of recurring headache such as a space-occupying lesion and intracranial aneurysm. It is a diagnosis of exclusion and history taking is pertinent, with the patient meeting the specified criteria per referenceable regional guidelines [1-6,9-15,16-19]. Although some people may present without migraineurs symptoms, the International Headache Society [1,2,4,10-15] defines migraine without aura as five or more bouts of headache lasting four to 72 hours that have gone untreated or have been unsuccessfully treated, plus any two of the following: (a) moderate to severe intensity, (b) throbbing or pulsing, (c) aggravated by movement, and (d) unilateral, plus any of the following: (i) nausea or vomiting; (ii) photophobia/phonophobia [1,2,4,10-15].

Several guidelines have also recommended using tools like the ID-Migraine™, Visual Aura Rating Scale (VARS), Migraine Disability Assessment (MIDAS) questionnaire, and Migraine-Specific Quality of Life Questionnaire (MSQ 2.1) to detect primary headache disorder [1-6,9-18]. Patients do not need to exhibit all the features listed in the International Classification of Headache Disorders, 3rd edition (ICHD-3) criteria; thus, a diagnosis of migraine can be made without characteristic symptoms such as throbbing or severe pain [1-6,9-18]. Following a comprehensive headache history and diagnosis of the original cause, neuroimaging and other ancillary tests such as electrocardiogram (ECG), erythrocyte sedimentation rate (ESR), and C-reactive proteins (CRP) are considered if other neurological etiologies of headache are suspected during physical or history evaluation [1-12,14-17]. Furthermore, validated screening methods, such as ID-Migraine™ tools, which factor in events like interference with activities, nausea, and sensitivity to light, might also be a rapid, practical first step in assisting doctors in identifying patients with migraine [1,4-6,11-18]. It is also important to consider other types of headaches that may present near similar to migraine. For example, tension and cluster headaches may be differentiated by their location, intensity, severity, duration of pain, concomitant symptoms, and behavior during an episode. Tension headaches often present with mild-to-moderate bilateral pain and may lack migraine-associated symptoms such as nausea [14-19]. Cluster headaches are characterized by large, intense unilateral pain, ipsilateral autonomic symptoms, and pain that lasts for more than three hours [14-19]. There are also other types of headaches with unique characteristics to be considered when evaluating patients with headaches.

With the burden of migraine and its attendant effects on human life, numerous pharmacological and non-pharmacologic treatment options are available; pharmacologic therapies have advanced over time, with significant breakthroughs made in the recent decade. Medications with higher adverse effects have been demoted, leaving less hazardous pharmacologic agents in the spotlight. When compared to earlier decades, the combination of newer innovations and safer drugs has improved migraine treatment outcomes [2,9-16]. However, as the search for more novel breakthroughs in migraine therapy continues, there is a need for a more individualized strategy to enhance treatment results by carefully evaluating modifiable risk factors that may inhibit good outcomes, such as excessive use of acute migraine drugs, ineffective therapy, obesity, smoking, excessive caffeine consumption, high-fat diets, anxiety, frequency of attacks, acute medication overuse, caffeine overuse, sleep disorders, and depression [1-6,9-20]. If undertreated or untreated, a migraine may lead to common complications that include migraine progression to chronic migraine, seizures, and strokes. These pharmaceutical advancements are examined using these case reports as an illustration for discussion.

## Case presentation

Case 1

The patient was a 39-year-old black female who came to the clinic with a history of recurring headaches that began a year prior to the time she presented and have occurred sporadically with no exact pattern, periodicity, or season, sometimes starting in the morning and other times during the middle of the day during peak hours of her job search. The patient said that the headache happened a few times a week or a few times a month at first but is now becoming more frequent, with more than three times every week. The patient reported her headache as starting on the left side of her head, extending frontally to behind her eyes, and then becoming "throbbing and sharp" around her head. It was graded 5-7/10 in severity, occasionally 8-10/10, and has steadily deteriorated. The patient also said that the headache lasts about two to four hours with varying intensities, relapsing and waning over the duration, and is accompanied by intermittent photophobia, which is considerably stronger when she drives on a bright day. The patient also mentioned associated nausea and a few incidents of vomiting in the past that usually preceded the headache or may occur simultaneously. She also reported occasional neck discomfort that starts a few minutes before the commencement of the headache and may remain throughout the episode or not at all. She also reported that laying down and not moving her head helps her headaches. They are also often alleviated by over-the-counter drugs such as Tylenol or ibuprofen and, at times, do not respond to her regular meds. She denied any other history of trauma or discomfort. She also denied dizziness, numbness, tingling, weakness, fever, neck stiffness, palpitation, shortness of breath, chest discomfort, cough, visual problems, loss of taste, smell, hearing, or any other neurological symptom, as previously indicated. Other reviews of systems were not contributory. The patient had no known allergies to any medications or items. Besides being jobless and actively seeking work, the patient denied any other recognizable life stressors but reported that the headaches have made life more difficult, interfering with her daily routine and focus. Sleep and psychiatric history were negative for any significant disorder. Her gynecological history was uneventful, and the patient denied any premenstrual symptoms or associations with her menses. The patient is married in a monogamous setting. Her mother died of breast cancer; there was no family history of migraine, and the other family history was negative. She smokes roughly three packs per day, has been a smoker for over 18 years, consumes wine occasionally, and does not use recreational drugs.

On examination, the patient was obese, with a body mass index (BMI) of 34.2 kg/m2. The head was atraumatic, normocephalic, and non-tender, and visual acuity and fundoscopy were normal. Inspection of the neck and palpation was normal. Her pulse rate was 87 bpm, blood pressure was 117/80 mmHg, respiratory rate was 16, oxygen saturation was 98%, and she was breathing at 16 cycles per minute, with normal air entry into the lungs bilaterally. There was no lymph node enlargement. The abdominal examination was normal. On neurological examination, the patient was oriented in place, person, and time; cranial nerves were intact, and there was no meningism. Her sensations were intact, and her deep tendon reflex was normal. Gait, finger-to-nose testing, Babinski, and Romberg were within the normal range. Other physical examinations were within the normal range. The patient's MIDAS score was 16 (moderate disability). Laboratory workups were also within the normal range, as shown in Table 1 below.

**Table 1:** Laboratory result panel

Laboratory test name	Test result	Reference range
Complete blood count		
Hemoglobin	13	11.5-17 g/dl
Red blood cell (RBC) count	5.3	4.1-5.6 x 10E6.uL
Packed cell volume	39	35-50%
Mean corpuscular volume (MCV)	96	80-98 fL
Mean corpuscular hemoglobin (MCH)	31.4	27-34 pg
Mean corpuscular hemoglobin concentration (MCHC)	33.4	32-36 g/dl
Red cell distribution width (RDW)	13.8	11.8-15.0%
Platelets	225	140-420 x 10E3/uL
Total white blood cell (WBC) count	6.5	4-10.5 x 10E3/uL
Neutrophil (absolute)	49	%
Lymphocytes	45	%
Monocyte (absolute)	5	%
Eosinophil (absolute)	1	%
Basophil (absolute)	0	%
Complete metabolic panel		
Glucose, serum	78	62-99 mg/dl
Calcium, total, serum	9	8.7-10.3 mg/dl
Sodium	140	134-145 mmol/dl
Potassium	4.1	3.5-5.1 mmol/dl
Chloride	103	96-106 mmol/dl
Carbon dioxide, total	21	18-29 mmol/L
Albumin	3.6	3.5-4.7 g/dl
Total protein	7.2	6.0-8.4 g/dl
Alkaline phosphate (ALP)	56	39-117 IU/L
Alanine transaminase (ALT)	25	0-32 IU/L
Aspartate aminotransferase (AST)	32	0-40 IU/L
Bilirubin	0.9	0-1.2 mg/dl
Blood urea nitrogen (BUN)	19	8-27 mg/dl
Creatinine, serum	0.8	0.58-1.0 mg/dl
BUN/creatinine ratio	21	12-27
Others		
Thyroid-stimulating hormone (TSH)	2.1	0.45-4.5 uIU/ml
Hemoglobin A1c	5.6	A1c < 5.7% = normal, 5.7% to 6.4% = prediabetes, and 6.5% or more = diabetes. Within the 5.7% to 6.4% = prediabetes range
Erythrocyte sedimentation rate (ESR)	19	0-27 mm/hr
C-reactive protein (CRP)	2.9	<3 mg/l
Urinalysis result		
Appearance	Turbid	
Color	Amber	
PH	6.5	
Protein	Negative	
Glucose	Negative	
Blood	Negative	
Ketones	Negative	
Bilirubin	Negative	
Lipid panel		
Total cholesterol	201	100-199 mg/dl
High-density lipoproteins (HDL)	44	30-75 mg/dl
Low-density lipoproteins (LDL)	70	0-99 mg/dl
Very low-density lipoprotein (VLDL)	40	4-41 mg/dl
Triglycerides	156	0-149 mg/dl

**Figure 1:**
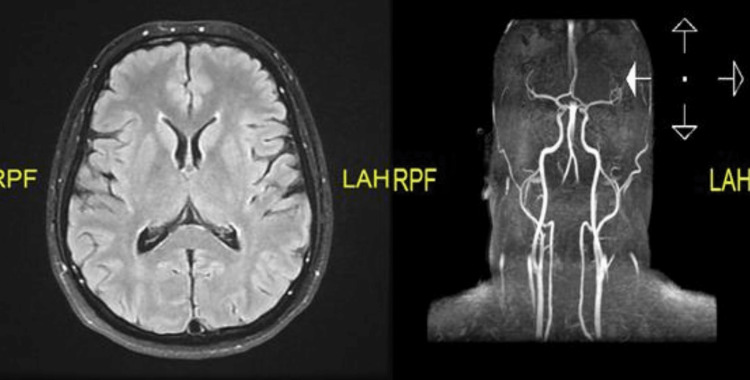
Axial FLAIR MRI of the brain at the level of the basal ganglia and MRA image showing normal findings FLAIR: fluid-attenuated inversion recovery; MRA: magnetic resonance angiography.

The patient had neuroimaging (see Figure 1 below) using magnetic resonance imaging (MRI) with magnetic resonance venography (MRV) and magnetic resonance angiography (MRA) to rule out intracranial mass lesion, white matter disease, dural venous sinus thrombosis, and aneurysms. Imaging findings were consistent with normal anatomical findings. There were no areas of intra- or extra-axial bleeds, no intra-cranial masses, no midline shift, no white matter disease, and no areas of infarction. The MRV images showed normal cerebral venous sinuses and jugular veins bilaterally, devoid of filling defects. The MRA images also showed no areas of stenosis, occlusion, or aneurysms in the anterior and posterior circulation. The chest X-ray came back normal. The ECG results were consistent with normal sinus rhythm and morphology. The patient was diagnosed with migraine with aura and was prescribed triptans. The patient had a lumbar puncture planned but declined the procedure. The patient was given sumatriptan 25 mg for abortive treatment with the intention of gradually increasing the dose, as well as topiramate for migraine prophylaxis. She was advised to avoid any triggering circumstances and to maintain a headache diary to precisely characterize the headache and its duration. In addition, the patient was sent to the neurology unit for additional evaluation. Five months later, the patient came to the clinic for a regular check-up with reports that her headaches had greatly improved, with less than two occurrences per month lasting shorter durations and allowing her to resume her normal everyday activities.

Case 2

The patient was a 21-year-old female driver with a migraine history who presented with a three-day history of worsening headaches that have been recurring for more than two years. In this index presentation, she described it as pounding in nature, rated about 7/10, usually starting in the temporal region of the head and progressing globally around the head, with occasional pulsations. She claimed that her headaches occur at any time, but most frequently when she is stressed at work. She described the headache as being associated with flashes of light or dazzling lights, progressing to an inability to look at flashes of light. She also reported pre-headache prodromes of increased food cravings, mood changes, restlessness, and anxiety, as well as difficulty concentrating for a few days or hours. During the headache episode, she experiences occasional vomiting and numbness on her forearms. Noise, movement, position, and light, according to the patient, aggravate her headache. She has seen several specialists, including a neurologist, and manages her migraines with acetaminophen, ibuprofen, and short-acting sumatriptan 50-100 mg two hourly as needed during acute attacks, with a satisfactory response and feeling joyful afterward. However, her headache at the time of presentation did not respond to her usual 50 mg of repeated sumatriptan doses, so she went to urgent care. She also mentioned that her headache has become more intense, prolonged, and frequent in recent weeks, with increased pulsations around her left eye. There was no significant family history of illnesses or past medical history other than her mother, who suffers from migraine. A psychiatric history revealed no significant concerns. The patient menstruates on a 28-day cycle, with occasional abdominal cramps but no headaches.

The patient was in acute painful distress, anicteric, not pale, had a BMI of 36, and not cyanosed upon examination. Aside from observable photophobia, a focused neurological examination revealed no findings. The fundoscopy results were normal. The patient was breathing normally in room air at 18 cycles per minute, with a pulse rate of 87 beats per minute and blood pressure of 120/80 mmHg. The chest examination was normal, with good air entry bilaterally. Other focused physical exams were normal. The laboratory workup was essentially normal, as shown in Table 2 below.

**Table 2:** Laboratory values

Laboratory test	Value	Reference range
Cholesterol	191	>200 mg/dl
High-density lipoprotein (HDL)	107	≥50 mg/dl
Triglyceride	87	<150 mg/dl
Low-density lipoprotein (LDL)	134	<100 mg/dl
Comprehensive metabolic panel		
Glucose	86	65-99 mg/dl
Blood urea nitrogen (BUN)	11	7-25 mg/dl
Creatinine	0.64	0.5-1.05 mg/dl
Glomerular filtration rate (GFR)	100	≥60 ml/min/1.73m^2^
BUN/creatinine ratio	17	6-22 (calc)
Sodium	139	135-146 mmol/L
Potassium	4.0	3.5-5.3 mmol/L
Chloride	103	98-110 mmol/L
CO2	28	20-32 mmol/L
Calcium	9.6	8.6-10.2 mg/dL
Total protein	7.4	6.1-8.1 g/dL
Albumin	4.8	3.6-5.1 g/dL
Globulin	2.6	1.9-3.7 g/dL
Albumin/globulin ratio	1.8	1.0-2.5 (calc)
Bilirubin	0.6	0.2-1.2 mg/dl
Alkaline phosphate (ALP)	59	37-153 U/L
Aspartate aminotransferase (AST)	19	10-35 U/L
Alanine transaminase (ALT)	15	6-29 U/L
Hemoglobin A1c	5.0	<5.7%
Others		
Thyroid-stimulating hormone (TSH)	3.35	0.40-4.50 mIU/L
Vitamin B12	600	190-950 pg/mL
Folate		2.7-17.0 (ng/mL)
Vanillylmandelic acid (VMA)	1.2	<6.8 mg/24 h
Complete blood count		
White blood cell (WBC)	4.6	3.8-10.8 thousand/uL
Red blood Cell (RBC)	4.60	3.80-5.10 million/uL
Hemoglobin	14.1	11.7-15.5 g/dL
Hematocrit	40.8	35.0-45.0%
Mean corpuscular volume (MCV)	88.7	80.0-100 fl
Mean corpuscular hemoglobin (MCH)	30.0	27.0-33.0 pg
Mean corpuscular hemoglobin concentration (MCHC)	34.6	32.0-36.0 g/dl
Red cell distribution width (RDW)	13.0	11-15%
Platelet count	293	140-400 thousand/uL
Neutrophil count	2420	1500-78000 cells/uL
Lymphocytes count	1748	850-3900 cells/uL
Monocytes count	368	200-950 cells/uL
Eosinophil count	32	15-500 cells/uL
Basophils count	32	0-200 cells/uL
Urinalysis		
Color	Yellow	Yellow
Appearance	Clear	Clear
Specific gravity	1.009	1.001-1.035
PH	6.0	5.0-8.0
Glucose	Negative	Negative
Bilirubin	Negative	Negative
Ketones	Negative	Negative
Occult blood	Negative	Negative
Protein	Negative	Negative
Urine drug test	Negative	Negative

**Figure 2:**
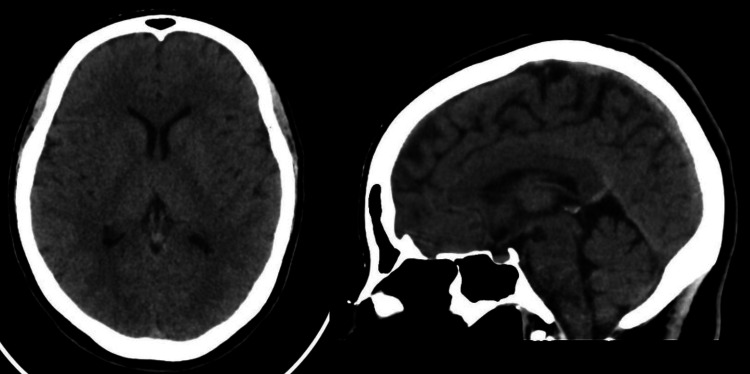
Axial non-contrast CT of the brain at the level of the basal ganglia and midline sagittal CT image showing normal finding

The patient previously had a chest X-ray, EKG, and several neuroimaging studies, but because this was a worsening headache, imaging studies were repeated and were essentially normal. A CT scan (see Figure 2 below) revealed no intracranial lesions. The urine drug test came back negative.

The patient was given intramuscular (IM) prochlorperazine 10 mg, IM ketorolac 60 mg, 18 mg dexamethasone, and galcanezumab after the headache subsided with a loading dose of 240 mg subcutaneously and 120 mg every month for the next five months. When the patient was seen several months later, she reported significant improvement in her symptoms' severity, duration, and frequency. She did, however, report itchiness and a rash at the injection sites that went away with time.

## Discussion

The pharmacologic approach in the patient's care plan was abortive therapy with triptans followed by prophylactic measures using topiramate. Pharmacologic management of migraine has evolved over the years. Breakthrough discoveries shaped this evolution in molecular theories of the pathophysiology of the complex neurovascular system that determines the presentations seen in migraine [2,11-17]. In many treatment guidelines, these management approaches are generally divided into drugs that are used in the acute relief of migraine attacks and those that prevent future occurrences [1-6,9-17]. While abortive therapies focus on stopping or reducing the intensity or progression of the headache, the preventive category aims to reduce the frequency of attacks. Table 2 below briefly summarizes some classes of medication in the two categories of management.

**Table 3:** Overview of typical pharmacological treatments a-n [1-6,11-17].

Class	Acute attack medications	Prophylaxis
Nonsteroidal anti-inflammatory drugs (NSAIDs)^a^	Simple analgesics, e.g., acetaminophen and ketorolac	Naproxen
Triptans^b^	Sumatriptan	
Ergot alkaloids^c^	Ergotamine	
Anti-dopaminergic^d^	Lasmiditan	
Antiemetics^e^	Metoclopramide, promethazine, prochlorperazine	
Opioids^f^		
Calcitonin gene-related peptide (CGRP) inhibitors^g^		Erenumab, atogepant, galcanezumab, eptinezumab, fremanezumab
5-HT antagonists^h^		Methysergide
Acetylcholine release blockers^i^		Botulinum toxin
Gamma-aminobutyric acid (GABA) inhibition enhancers^j^		Gabapentin, valproic acid
Central inhibitors (tricyclic antidepressants, beta-blockers)^k^		Amitriptyline, nortriptyline, extended-release propranolol, timolol, atenolol
Voltage channel regulators (calcium channel blockers)^l^		Verapamil
Complementary or alternate therapy^m^	Butterbur, vitamin B2, CoQ10, melatonin, yoga, acupressure, acupuncture, biofeedback, massage	Butterbur, vitamin B2, CoQ10, melatonin, yoga, acupressure, acupuncture, biofeedback, massage
Device^n^	Transcutaneous electrical nerve stimulation, vagus nerve stimulators, smartphone-controlled electronic pulses to relieve devices	Transcutaneous electrical nerve stimulation

Triptans, nonsteroidal anti-inflammatory drugs (NSAIDs), topiramate, and the newer monoclonal antibodies (mAbs) were prescribed to these patients based on local availability and best practices, frequency of attacks, the physicians' therapeutic knowledge of the risk profile of the selected medicine, and other comorbidities such as BMI and smoking status. Several factors determine the precise pharmacological choice in each situation [1,2,4-6,9-17,20-24], including the nature and severity of the migraine, the presence and severity of comorbidities, the most expedient route of administration at presentation (transdermal, intranasal, intravenous, intramuscular, or oral), safety and side effect profile, and, most importantly, the patient's treatment response. Furthermore, some writers and recommendations have advocated a stratified and personalized therapy strategy [1,2,4-6,9-17,20-24].

Overview of mechanism of abortive therapies

In the first patient in case 1, the patient presented in acute phases with abortive therapy as the first line of management. Triptans and NSAIDs are amongst the most common first-line medications in outpatient and emergent settings [1-5,23-25]. The efficacy of these breakthrough migraine medications like NSAIDs, triptans, and placebos has been analyzed in various validated meta-analyses. In a meta-analysis by Xu et al. [25] examining the treatment effectiveness of migraine with this acute medication in 2016, they reviewed 88 double-blinded placebo-controlled randomized trials of 39,004 participants between 1998 and 2016. Their outcome measures showed that all treatments were more effective than placebo, with statistical significance in their pain-free measures. In their pairwise stratified analysis, the triptans showed better efficacy than the NSAIDs, with sumatriptan the least effective amongst the triptans. However, the NSAIDs like ibuprofen and aspirin showed milder adverse events than triptans, and their findings were replicable in other homogenous analyses. The combination of triptans and NSAIDs in a multi-component medication has also been shown to be more effective in two-hour pain-free outcomes than single medication use [1-6,22-25]. Ergot amines and opioids are generally regarded as second-line rescue therapies. They have shown evidence of effectiveness but more side effects [1-6,22-25]. Multiple acute interventions for the treatment of episodic migraine were evaluated in 15 systematic reviews and 115 randomized clinical trials involving 28,803 participants with migraine headaches; pain relief, pain-free, and adverse events were determined. Juliana and her team [26] concluded that, among other things, while ergotamine showed effectiveness, evidence supporting opioid were limited [1-6,22-25]. Several other similar analyses have been consistent for these second-line therapies [1-4,20-28].

Overview of mechanism of prophylaxis therapies

The patient in case 1 responded to topiramate, as evidenced by a reduced frequency of migraine attacks and improved quality of life. A variety of medications, including beta-blockers, calcium channel blockers, antiepileptics, NSAIDs, and antidepressants, have shown promise as preventative treatments for migraines [23-25]. In a comparative effectiveness analysis by Jackson et al., in 2015, [26] on randomized controlled trials in 15,493 patients with migraine greater than four weeks, pooled results showed overall superiority of these medications (levetiracetam, topiramate, enalapril, captopril, valproic acid, and amitriptyline) over placebo. They further stratified their analyses to examine the comparative effectiveness among the prophylactic medications. Their result also showed that amitriptyline was more effective than many other drugs like topiramate, venlafaxine, or propranolol. Each of the classifications has its unique side effect, but these network studies showed no difference amongst the classes in the likelihood of experiencing side effects, and the selection of these medications must consider their individual risk profiles, patient's comorbidity, and other personalized treatment strategies [1-6,9-16,20-26].

Beta-blockers are clearly effective in migraine prophylaxis and are very well studied in a lot of placebo-controlled, randomized trials. The best evidence has been obtained for metoprolol and propranolol [20-30]. Also, bisoprolol, timolol, and atenolol might be effective, but the evidence is less convincing compared with propranolol and metoprolol [20-30]. In a double-blind, cross-over study, the effect and tolerance of the non-selective beta-blocker propranolol in a dosage of 80 mg twice daily was compared to that of the beta 1-selective beta-blocker metoprolol 200 mg once daily in Durables(r) (a controlled-release formulation) [25]. The attack frequency, migraine days, severity score, consumption of acute medication, and subjective evaluation were the main parameters used for evaluation. Thirty-six patients with classical or common migraine were included. Thirty-three completed the investigation. It is concluded from the results that there are no differences in efficacy between metoprolol and propranolol regarding the studied parameters. Both drugs reduced the migraine symptoms compared to the run-in period and were generally well tolerated. Numerous other placebo-controlled, randomized studies have shown that beta-blockers are helpful in migraine prevention [10,20-29]. The impact and tolerance of these beta-blockers have been studied and recommended by several local and international guidelines [27-29]. Although most of these studies were done decades ago, their results still remain relevant in today's world. Furthermore, inhibitory molecules of riboflavin or magnesium, or the use of botulinum toxin to stop the release of acetylcholine in presynaptic channels, have also been used in this preventative strategy to deal with oxidative or hyperexcitability processes in neurons and have shown superiority over placebo [1-6,30,31].

Recent advances

In case 2, the patient responded to the newer mAbs, which is consistent with the numerous recorded successes with the use of these newer drugs in the treatment of migraine. One of those advances is in the discovery of receptors and molecules that play a part in pathophysiology. Mutations in TREK (a potassium background channel with two pore domains that is widely distributed in the nervous system) gene expression, a gene that encodes instructions for a subset of potassium ion channels, were discovered in 2010 by some researchers [3,31-37]. Potassium channels are responsible for regulating nerve cells' resting membrane potentials, and mutations in them may lead to overexcitation resulting in response to lower pain levels [3,31-37] and/or CGRP. Targeting TREK activation/inhibition is being investigated as potential effectiveness in migraine therapy [3,31-37].

**Figure 3:**
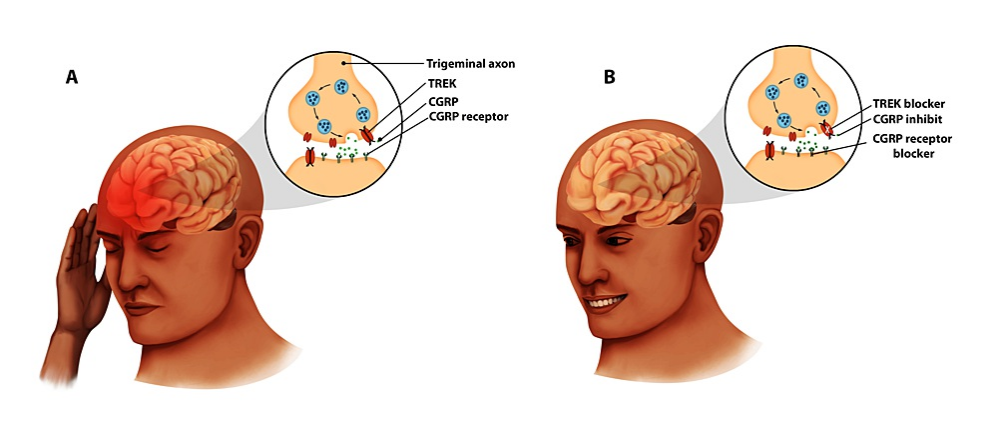
Illustration of headache and the interplay between the CGRP receptors and blocker The image concept was adapted from [15,16]. (A) Headache in a patient before CGRP antibody. (B) Post-CGRP blocker and CGRP receptor blocker. Legal protection for original work. Both the artwork and the accompanying words were originally developed by us. CGRP: calcitonin gene-related peptide.

Previously, migraines were assumed to originate from peripheral blood vessel dilatation. Nonetheless, newer studies suggest that it may be caused by a malfunction in the brain stem area that controls pain sensation and blood vessel tone [34-38]. This new postulation suggests that triggers such as certain foods, stress, or hormones frequently start a series of events [34-39]. These stimuli now produce dysregulation in the part of the brain stem that governs vascular tone, resulting in peripheral vasodilation. The dilated vessels then trigger stretch signals and impulse transmission to the brain stem, particularly from trigeminal neurons. This impulse transmission results in the production of inflammatory and vasoactive chemicals such as interleukins and CGRP [17,34-38], which produce further dilatation. Further dilatation will result in more inflammatory responses with increased vascular permeability, which will ultimately lead to mast cell activation and tissue edema, and the cascade will continue as the attack continues [1,32-39]. More specifically, when the neurons in the trigeminal ganglia are stimulated, they produce inflammatory chemicals like CGRP. Recent years have seen a rise in the use of CGRP inhibitors as a preventative treatment for migraine [17,34-39]. Trigeminal neurons that generate A-fibers and C-fibers contain CGRP receptors and CGRP. A few instances were used to illustrate the unique involvement of CGRP, among other vasoactive chemicals, in migraine. For example, CGRP is released into the cerebral venous outflow during cluster headaches and acute migraines [36-42]. Furthermore, in migraine sufferers, intravenous CGRP may trigger migraine-like symptoms [36-42]. These discoveries prompted the creation of anti-migraine medications that block CGRP function [36-42]. Following roughly two decades of well-controlled studies, the FDA approved these cloned anti-CGRP IgG mAbs like fremanezumab, erenumab, and galcanezumab for chronic migraine prophylaxis [36-42], and in 2019, ubrogepant, a selective CGRP receptor blocker for the acute treatment of migraine with or without aura [36-42]. As the usage of these newer treatments grows in the general population, scientists are rigorously scrutinizing the stated effectiveness in comparison to the general population sample effect. In a meta-analysis by Deng and his team in 2020 on 4402 patients enrolled in 11 heterogeneous randomized placebo-controlled clinical trials with similar outcome measures, they sorted to examine the efficacy of CGRPs compared to placebo [40]. CGRP resulted in an improvement in the 50% responder rate, a decrease in the monthly migraine rate, and a decrease in the use of acute migraine medications [40]. Some other researchers used a likely to help vs. harm analysis to compare the effectiveness and absolute difference in risk/benefit ratio of anti-CGRP mAbs, propranolol, and topiramate for the prevention of chronic migraine [34-36,41-44]. There is widespread consensus that the benefit-risk profile of anti-CGRP mAbs is superior to that of currently available therapies for both episodic and chronic migraine [33-36,41-46]. Several more studies and randomized controlled trials have also corroborated the present body of data on the efficacy and safety of CGRP mAbs for the treatment of episodic migraine [34-36,41-45]. In comparison to propranolol and topiramate for the treatment of episodic migraine and the prevention of chronic migraine, respectively, all dosages of anti-CGRP mAbs showed a greater Likelihood to help values [34-36,41-45]. When compared to the mechanism of action of CGRP antagonists, the mechanism of action of older migraine treatments is not as well understood. CGRP antagonists were created based on the understanding that inhibiting the involvement of CGRP in the cascade of events is effective in treating migraines [34-36,41-45]. Figure 3 below provides a "literal" illustration of the relationship between CGRP inhibition and the alleviation of migraine symptoms.

Recently, another group of pharmacologic drugs also came into the spotlight, with the pharmaceutical agent tonabersat serving as an illustrative case in point [44-46]. Inhibitions of neurovascular gap junctional communications between glial cells of the trigeminal ganglion and neurons have been the focus of tonabersat, a novel benzopyran-based molecule that aims to reduce cortical spreading [44-46]. Tonabersat was shown to be beneficial in preventing migraines with aura in a randomized, double-blind, controlled study of 39 patients; however, it has not yet been approved by the FDA [44-46] at the time of this review but has been added in the limitless potentials in the management of migraines in these new decades. There have also been efforts to inhibit or modulate the effects of other molecules involved in neurotransmission. For example, serotonin receptors, nitric oxide synthase, prostaglandin E receptors, and N-methyl-D-aspartic acid (NMDA) receptors for glutamate [1-6,16,17].

## Conclusions

Recent developments in novel genetic findings have increasingly dispelled the mystery surrounding migraine. This case series and the discussions highlight the most recent developments and trends in pharmacologic treatments for migraine. In agreement with all other recent findings, our patients responded to both the existing and novel medications. Recently, synaptic and axonal inhibitions have made headlines, with CGRP and CGRP receptor inhibition prominent examples. These newer medications' mechanisms have created a flood of interest in migraine treatment research.
